# An extended dose–volume model in high dose‐rate brachytherapy – Using mean‐tail‐dose to reduce tumor underdosage

**DOI:** 10.1002/mp.13533

**Published:** 2019-05-15

**Authors:** Björn Morén, Torbjörn Larsson, Åsa Carlsson Tedgren

**Affiliations:** ^1^ Department of Mathematics Linköping University SE‐58183 Linköping Sweden; ^2^ Radiation Physics, Department of Medical and Health Sciences Linköping University SE‐58183 Linköping Sweden; ^3^ Medical Radiation Physics and Nuclear Medicine Karolinska University Hospital SE‐17176 Stockholm Sweden; ^4^ Department of Oncology Pathology Karolinska Institute SE‐17176 Stockholm Sweden

**Keywords:** cold volumes, CVaR, dose–volume model, dosimetric index, dwell time optimization, EUD, mean‐tail‐dose, TCP

## Abstract

**Purpose:**

High dose–rate brachytherapy is a method of radiotherapy for cancer treatment in which the radiation source is placed within the body. In addition to give a high enough dose to a tumor, it is also important to spare nearby healthy organs [organs at risk (OAR)]. Dose plans are commonly evaluated using the so‐called dosimetric indices; for the tumor, the portion of the structure that receives a sufficiently high dose is calculated, while for OAR it is instead the portion of the structure that receives a sufficiently low dose that is of interest. Models that include dosimetric indices are referred to as dose–volume models (DVMs) and have received much interest recently. Such models do not take the dose to the coldest (least irradiated) volume of the tumor into account, which is a distinct weakness since research indicates that the treatment effect can be largely impaired by tumor underdosage even to small volumes. Therefore, our aim is to extend a DVM to also consider the dose to the coldest volume.

**Methods:**

An improved DVM for dose planning is proposed. In addition to optimizing with respect to dosimetric indices, this model also takes mean dose to the coldest volume of the tumor into account.

**Results:**

Our extended model has been evaluated against a standard DVM in ten prostate geometries. Our results show that the dose to the coldest volume could be increased, while also computing times for the dose planning were improved.

**Conclusion:**

While the proposed model yields dose plans similar to other models in most aspects, it fulfils its purpose of increasing the dose to cold tumor volumes. An additional benefit is shorter solution times, and especially for clinically relevant times (of minutes) we show major improvements in tumour dosimetric indices.

## 1 Introduction

High dose–rate brachytherapy (HDR BT) is a modality of radiation therapy used for cancer treatment. In HDR BT, the radiation is delivered from within the body using applicators and/or catheters (hollow needles) which are temporarily inserted, close to or within the tumor, to guide a small, sealed photon‐emitting source of ionizing radiation. At treatment delivery, the single radiation source, commonly of the isotope ${}^{192}Ir$, is driven through the catheters, programmed to rest in the selected dwell positions along the catheters for predetermined dwell times (corresponding to bixels in external beam radiation therapy, EBRT). The combination of dwell positions and the dwell times in these determines the dose distribution within the patient’s body. The task of planning is performed before treatment delivery during the dose planning stage. See Fig. Fig. 1 for an overview of the HDR BT treatment process.

**Figure Fig. 1:**
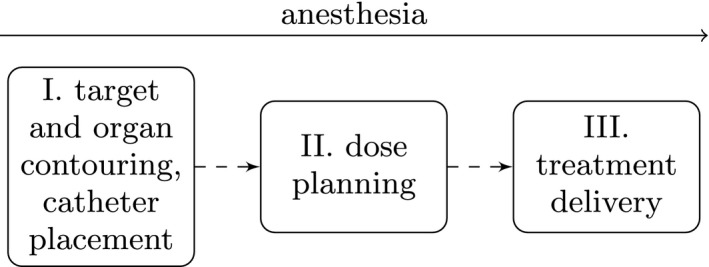
Overview of the HDR BT treatment process. The first step (I) is image‐guided insertion of catheters and outlining of target and organs on images. The second step (II) is the dose planning, in which a large part is to decide the dwell times to obtain a good dose distribution for the anatomy of the patient. The final step (III) is the treatment delivery. All steps are typically performed while the patient is under some form of anesthesia.

In HDR BT, the positions of the catheters are determined first, followed by the dwell time pattern. The roles of the catheters and the dwell time patterns in these are the HDR BT equivalent to the fields and fluence maps in EBRT. Clinically used treatment planning systems offer both graphical tools and automated algorithms which are based on optimization (such as IPSA^1^ and HIPO^2^) to perform the dose planning. The dose plan evaluation, according to clinical treatment guidelines, is based on the dose–volume histogram (DVH) concept and the so‐called dosimetric indices that are derived therefrom, see Ref. 3 for HDR BT guidelines for prostate cancer. The automated methods available today are based on linear penalties and have been shown to possess weaknesses such as producing fewer and longer dwell times than manual methods,^4^, ^5^ and to correlate weakly with the dosimetric indices.^6^ Furthermore, the automated methods based on linear penalties require the user to calibrate penalty parameters which can be a difficult and time consuming task.

The above‐mentioned weaknesses of current methods have resulted in a research focus on finding improved automated methods for dwell time optimization, capable of using the dosimetric indices (or an approximation thereof) as the actual quality measures for the optimization.^7^, ^8^, ^9^, ^10^ While the advantage of using the dosimetric indices as quality measures is obvious (due to the clinical guidelines), it is important to be aware of the pitfalls and weaknesses of this approach, which can be severe if not foreseen. In the DVH‐based models that have been proposed a weakness is that the dose to the coldest (least irradiated) volume of the tumor is not taken into account. (This volume is not necessarily contiguous, which is how the expression coldest volume is used in the following.) To give an example, if the objective is to maximize the lowest dose received in 95% of the tumor, an optimization algorithm will strive solely to fulfil this aim and will not, unless explicitly instructed, consider the dose to the remaining 5% of the volume. If necessary, it will sacrifice (reducing the dose to) the 5% of the tumor that receives the lowest radiation dose, even for a negligible gain in its objective.

The aim of this work is first to develop an improved and safer DVH‐based optimization model for dose planning. Our starting‐point is published DVH‐based models^7^, ^9^ that have been evaluated positively against clinical dose plans.^8^, ^10^ However, our work offers no comparison with such plans but focuses solely on the extension of the model. Published DVH‐based models are extended by the addition of a component to mitigate severe underdosage to the part of the target that receives a dose that is lower than the prescription level; the added component works as a safeguard against such underdosage. Secondly, the aim is also to carefully describe the reasoning leading up to our suggested remedy and to contribute to an increased awareness and understanding of possible pitfalls in a straightforward use of mathematical optimization methods for DVH‐based models.

## 2 Problem formulation

Like other radiotherapy modalities, HDR BT is today planned using three‐dimensional, anatomical patient information derived from images on which target and organs are contoured (see Fig. Fig. 1). The goal of the treatment is local tumor control balanced with an acceptable risk of normal tissue complications. Tumor control is (most likely) achieved by delivering a sufficiently high dose to the planning target volume (PTV), which is the tumor with an extra margin. The healthy organs and tissues [organs at risk (OAR)] present in the proximity of the PTV should be spared, if possible, to reduce the risk of complications. The contoured PTV and OAR are in the following technical context referred to as structures. For the sake of the dose planning, the structures are represented as dose points (corresponding to voxels in EBRT), where the absorbed doses (in Gray, Gy) to the tissue in these small volumes (in BT approximated by values of absorbed dose in water) are calculated^11^ and used for the construction and evaluation of dose plans. For an introduction to radiation therapy and HDR BT see, for example, Ref. 12.

An important tool for the dose plan evaluation is the (cumulative) DVH. For each level of radiation dose, the DVH states how large a portion of the PTV (or OAR) that receives at least (or at most) this level of radiation. One such point on the DVH curve is called a dosimetric index (DI) and corresponds to two types of indices, $V_{x}^{s}$ and $D_{y}^{s}$. The index $V_{x}^{s}$ is the portion of the volume of structure *s* that receives at least *x*% of the prescribed dose if *s* is the PTV, or receives at most *x*% of the prescribed dose if *s* is an OAR. The index $D_{y}^{s}$ is the smallest dose that is received by the *y*% of the volume of structure *s* which receives the highest dose. Figure Fig. 2 shows an example of a DVH curve and indices $V_{x}^{s}$ and $D_{y}^{s}$. Guidelines^3^ for HDR BT for prostate cancer recommend that $D_{90}^{\mathrm{PTV}}\;\geq \;100\%$ of the prescription dose and that $V_{100}^{\mathrm{PTV}}$ is at least 95%. In the following, if no structure is specified, $V_{x}$ and $D_{y}$ refer to the PTV.

**Figure Fig. 2:**
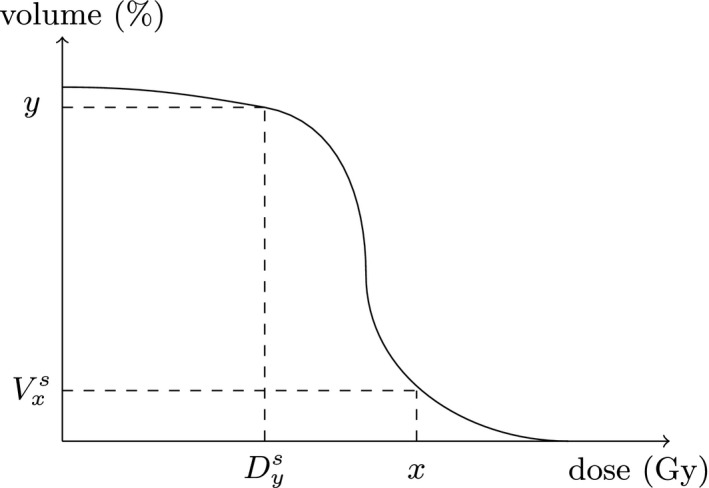
Example of a dose‐volume histogram.

Because of the central role of DIs in dose plan evaluation, according to the clinical treatment guidelines, optimization models that explicitly include them are of high interest and have been a topic of recent research. To mathematically model the DIs, a Heaviside step function is used for each dose point to indicate whether the dose is high enough (for PTV) or low enough (for OAR). Each PTV or OAR Heaviside function can be modeled using a binary indicator variable, taking the value one if the criterion is satisfied and zero otherwise. A model based on DI criteria yields a mixed‐integer program (MIP) and such a model is referred to as a dose–volume model (DVM). A DVM for HDR BT was first proposed by Bëlien et al.^13^ Models of this type were further studied by Siauw et al.^7^ and Gorissen et al.^8^ Because these models are computationally hard to solve to optimality, heuristics are often used to find good solutions to practical instances.^7^, ^9^, ^13^ (Heuristics are algorithms with no guarantees of finding an optimal solution and no information about the quality of an optimal solution, but which in practice often are able to quickly find good solutions.) The DVM formulation developed by Guthier et al.^10^ approximated the Heaviside functions with smooth, nonconvex functions and find good solutions in short solution times (a few seconds). With their approximations there are, however, no guarantees of optimality or even feasibility in dosimetric index constraints.

The DIs are in clinical practice mainly used in the evaluation of dose plans and DVMs are not commonly used in clinically available treatment planning software. The DVMs have, however, been shown to produce dose plans which are clinically acceptable.^8^, ^10^ Reference 8 includes the opinion from an expert on the dose plans, and Ref. 10 compares dose plans from a number of optimization models, including models based on dose–volume criteria, with dose plans from manual planning.

The dosimetric index $V_{x}^{s}$ is a rather rough way of evaluating a dose plan. Because the contribution to a DI (which is based on the discontinuous Heaviside step function) from each dose point is either zero or one, small variations in the dose distribution can have a large effect on the DI. For example, if the prescription dose is 10 Gy, anything above 10 Gy is considered equally acceptable while anything below 10 Gy is equally poor, with no differentiation made between values that are below 10 Gy. Further, two dose distributions can have very different DVH curves but the same value of several DIs or vice versa. In particular, even if a DI requirement for the PTV is satisfied there might be volumes of the PTV where the dose is much too low for the intended treatment effect.

This adverse property of a DI for the PTV constitutes a weakness in any DVM, because in such models, the dose to the underdosed volume of the PTV is not in any way considered in the optimization model, as long as the specified DIs are satisfied. Hence, there is a risk that the treatment effect is lower than intended, and lower than what is indicated by only the DI. This is due to the fact that optimization models only take explicit aspects into account and that there is no implicit consideration of other aspects of a good solution.

DIs are examples of evaluation criteria that consider solely physical dose, while radiobiological effects of the dose distribution are captured only indirectly. An example of an index that is based on a radiobiological model is the tumor control probability (TCP). The TCP estimates the probability of local tumor control, which is the probability that no malignant cell survives the radiation dose.

Tomé and Fowler^14^ studied the effect on TCP when volumes of the PTV, of various sizes, received a lower dose than the prescription dose. They found that underdosage has a large impact on TCP, even when the underdosed volume was as small as 1% of the PTV. They also found that this was still the case when 80% of the PTV received a 10% boost in addition to the prescription dose. Their conclusion is that it is not enough to specify a prescription dose, which should be received by, for example, 95% of the PTV unless some additional means ensure the dose to the coldest volume of the PTV to be high enough.

The aim of this paper is to extend a DVM model to also take the dose to the coldest volume of the PTV into account. This is motivated by the fact that underdosage to parts of the volume of the PTV can be present even though DI requirements are fulfilled and the above mentioned observation of Tomé and Fowler.^14^ For our extension, we consider the mean dose to the coldest volume of the tumor, also referred to as cold mean‐tail‐dose.

An outline for the remainder of the paper is as follows: the complete optimization model and its settings are presented in Section 3, results and analyses are given in Section 4 and discussed in Section 5, and finally conclusions are given in Section 6.

## 3 Materials and methods

A measure that is related do the DVH and the DIs is the conditional value‐at‐risk (CVaR), which was introduced as a financial measure for risk assessment.^15^ In finance, CVaR is the expected loss for the *α*% worst outcomes, while in radiation therapy the interpretation is the mean dose to a portion *α* of a volume. In the context of radiation therapy, CVaR has been referred to as mean‐tail‐dose. For our purpose, we are interested in the portion that receives the lowest dose, comparable with the worst outcomes. For a specified portion (1−*α*)% of the volume, CVaR${}_{\left(1-\alpha \right)}$ quantifies the mean dose to the (1−*α*)% of the volume that receives the lowest dose. It has been shown that CVaR can be modeled with linear expressions,^15^ either to maximize (or lower bound) the mean dose to a specified portion of the volume that receives the lowest dose or to minimize (or upper bound) the mean dose to a specified portion of the volume that receives the highest dose. In intensity‐modulated radiation therapy (IMRT), Romeijn et al.^16^ were the first to formulate a model for dose planning with CVaR constraints; a recent study in IMRT with CVaR constraints can be found in Ref. 17. An optimization model for HDR BT with CVaR constraints is proposed in Ref. 18.

The following optimization model for dose planning is adopted from the DVMs in Refs. 7 and 9, with an additional CVaR component for the PTV added to the objective function. The CVaR component is added to explicitly address the identified weakness of the DVM. CVaR has previously been used as a convex approximation of dosimetric indices.^16^ Worth noting is that the criteria $V_{100}$ and CVaR are not in conflict. The DVM in Ref. 9 contains an additional dwell time modulation restriction component which we omit. The inclusion of dwell time modulation restriction is common to mitigate the long dwell times occurring with linear penalty models.^5^ However, whether such restrictions are beneficial or not is a complex question, which is analysed in, for example, Ref. 19.

The indices, sets, parameters and variables that are used in the optimization model are introduced in Table 1.

(1) $$\max\;\;w_{1}\left(\frac{1}{\left|T\right|}\sum_{i\in T}y_{i}\right)+w_{2}\left(\zeta^{\alpha }-\frac{1}{\left(1-\alpha )|T\right|}\sum_{i\in T}\eta_{i}\right)$$

(2) $$\begin{array}{cc} & \text{subject to}\\ & \sum_{j\in J}d_{\mathrm{ij}}t_{j}\geq Ly_{i},\;\;\;i\in T \end{array}$$

(3) $$\sum_{j\in J}d_{\mathrm{ij}}t_{j}\leq U^{s}+M^{s}\left(1-v_{i}^{s}\right),\;\;i\in OAR_{s},s\in S$$

(4) $$\sum_{i\in OAR_{s}}v_{i}^{s}\geq \tau^{s}\left|OAR_{s}\right|,\;\;s\in S$$

(5) $$\eta_{i}\geq \zeta^{\alpha }-\sum_{j\in J}d_{\mathrm{ij}}t_{j},\;\;i\in T$$

(6) $$\eta_{i}\geq 0,\;\;i\in T$$

(7) $$y_{i}\in \left\{0,1\right\},\;\;i\in T$$

(8) $$v_{i}^{s}\in \left\{0,1\right\},\;\;i\in OAR_{s},s\in S$$

(9) $$t_{j}\geq 0,\;\;j\in J$$

**Table 1:** Indices, sets, parameters and variables used in the optimization model

Indices	
*i*	Index for dose points
*j*	Index for dwell positions
*s*	Index for OAR
Sets	
*T*	Set of dose points in the PTV
*S*	Set of OAR
$OAR_{s}$	Set of dose points in OAR *s*,* s* ∈ *S*
*J*	Set of dwell positions
Parameters	
$d_{\mathrm{ij}}$	Dose rate contribution from dwell position *j* ∈ *J*, to dose point $i\in T\cup (\cup_{s\in S}OAR_{s})$
*L*	Prescription dose to the PTV
$U^{s}$	Upper dose bound for OAR *s*,* s* ∈ *S*
$M^{s}$	Maximum excess dose (above $U^{s}$) to OAR *s*,* s* ∈ *S*
$\tau^{s}$	A portion of the volume of OAR *s*,* s* ∈ *S*, that should satisfy the dose bound, $U^{s}$
*α*	A portion of the volume of the PTV
$w_{1}$	Non‐negative weight for $V_{100}$
$w_{2}$	Non‐negative weight for CVaR
Variables	
$y_{i}$	Indicator variable for dose point *i* ∈ *T*, which equals one if the dose is at least *L*, and is zero otherwise
$v_{i}^{s}$	Indicator variable for dose point $i\in OAR_{s},\;s\in S$, which equals one if the dose is at most $U^{s}$, and is zero otherwise
$\zeta^{\alpha }$	Auxiliary variable used for finding the CVaR value
$\eta_{i}$	Auxiliary variable for the maximum of two values
$t_{j}$	Dwell time in dwell position *j* ∈ *J*

Here, |·| denotes the cardinality of a set. The objective, given in formulation (1), is to maximize the weighted sum of the value of DI $V_{100}$ for the PTV and the CVaR${}_{\left(1-\alpha \right)}$ value. The indicator variable for each dose point in the PTV equals one if the dose is high enough, and zero otherwise, and is defined by constraint (2). The first part of the objective function is thus the portion of the PTV (dose points) receiving at least the prescription dose. For OAR, the DIs are defined by constraints (3) and (4), where the first constraint ensures that each indicator variable takes the correct value, while the second imposes a lower bound on the DI, that is, on the portion of the OAR which receives a dose that is low enough. This mathematical formulation of the DIs is used in Ref. 9.

The CVaR component of the model consists of the second term in the objective function (1), in which the auxiliary variables $\eta_{i},\;i\in T$, are defined by constraints (5) and (6), which are a linear formulation of the definitional constraint $\eta_{i}\;=\;\max\left(0,\zeta^{\alpha }-\sum_{j\in J}d_{\mathrm{ij}}t_{j}\right)$, *i*  ∈  *T*. The auxiliary variable $\zeta^{\alpha }$ takes the value of the highest dose that is received among the (1−*α*)% dose points receiving the lowest dose. Variables $\eta_{i}$, *i* ∈ *T*, equal the dose deficits compared to the value $\zeta^{\alpha }$ (if positive). Hence, the second term in the objective function is the mean dose that is received by the (1−*α*)% that receives the lowest dose. This (standard) formulation of CVaR is the same as in Romeijn et al.^16^

To give an example on how $V_{100}$ and CVaR are calculated, assume that we have a dose distribution consisting of ten dose points receiving 5, 7, 8.5, 8.5, 8.5, 10, 12, 13, 15, and 17 Gy, with 8.5 Gy as the prescription dose. Then the value of $V_{100}$ is 80% and the value of CVaR${}_{20}$ is 6 Gy (mean value of the 20% dose points receiving the lowest dose).

In the remainder of this section, we introduce the data and the settings that we have used for the computer simulations. We have tested our model’s performance against the DVM retrospectively on clinical implants, using contoured PTV, OAR and catheter placement information from ten patients earlier treated for prostate cancer. The number of dose points in the optimization models was in the range 4369–7939 and distributed according to Ref. 20, while the number of dose points for the evaluation of dose plans was in the range 51 974–134 509 and distributed uniformly with a volume of $1\;{\text{mm}}^{3}$ per dose point. (The latter sets of dose points were used for calculating dosimetric indices and all other studied quantities.) The number of dwell positions was in the range 190–352, and the number of catheters in the range 14–20. On the medical images, the PTV, urethra, and rectum were already outlined, according to the practice of the clinic that provided the patient data. In addition, according to the standard procedure, we added artificial, healthy tissue surrounding the PTV.

Gurobi (Gurobi Optimization, Inc., Houston, USA) version 7.0.1^21^ is a state‐of‐the‐art software for linear optimization problems. We used its implementation of the simplex algorithm for solving all linear programs (see Ref. 22) and its branch‐and‐bound implementation for solving all MIPs (see Ref. 23). The computer simulations were performed on a PC with an Intel(R) Core(TM) i7‐6500U CPU, 2.50 GHz processor, 16 GB RAM, and a 64‐bit Windows 10 operating system.

In Table 2, we introduce the models that we compare. Model DVM is the standard DVM (in the actual implementation, the CVaR constraints and variables are not included at all), while the mean‐tail dose model (CVaR) has only the CVaR component in the objective function. The objective of the dose‐volume mean‐tail‐dose model (DVMCVaR) includes both the $V_{100}$ component and the CVaR component, the purpose of the latter being to increase the dose to the coldest volume. We found that the specific choice of value for $w_{2}$ in this model had no significant impact. The solution progress was very similar for all weights $w_{2}\;>\;0$ that we tried and thus it is unlikely that any particular tuning of the weights is necessary. Model MTDM is included because it gives an upper bound on how much the dose to the coldest volume can be improved.

**Table 2:** Specification of the tested models with corresponding objective function weights

Model	$w_{1}$	$w_{2}$
DVM	1	0
DV‐MTDM	1	1
MTDM	0	1

The values used for the dose bounds and the parameters for the DI are given in Table 3. For CVaR, we chose 1−*α* = 1% because it was shown in Ref. 14 that even such a small volume with a too low dose could have a significant impact on the TCP. In the following, we drop the subscript on CVaR, because the portion 1−*α* is fixed (to 1%).

**Table 3:** Values used for prescription dose and dose bounds. Values for planning target volume (PTV) and organs at risk are adopted from Deist and Gorissen.^9^

Volume	Dose target (Gy)	Dose bound (Gy)	Portion (%)
PTV	8.5		
Urethra	10.0	10.6	90
Rectum	7.2	8.0	90
Artificial tissue		8.5	

To get an indication of the effect from cold volumes in the PTV we have used TCP in the analysis. For the calculation of TCP, we implemented the algorithm in Ref. 24 with the parameters values:^14^ $N_{C}\;=\;8.423\;\times \;10^{8}$, $\sigma_{\mathrm{pop}}\;=\;0.135$, $\sigma_{\mathrm{ind}}\;=\;0.045$, and $SF_{2}\;=\;0.48$. Further, the parameter *α*/*β* was set to 1.5, which is a suggested value for prostate cancer.^25^

A full prostate cancer treatment typically includes both EBRT and two sessions of HDR BT. To simulate a full treatment, we have rescaled the absolute dose values in the TCP calculation by a constant factor, so that a TCP value of 95% corresponds to a uniform dose distribution equalling the prescription dose. The calculated TCP values should thus not be seen as an exact representation of the treatment effect, but rather as a means for comparing dose plans. (The use of TCP has been discussed^26^, ^27^ and there is both a need for better estimates of parameter values and for more clinical studies on the correlation with the treatment outcome.)

The equivalent uniform dose (EUD), also known as the generalized mean dose, is another measure of the biological effect from a dose distribution. It is meant to summarize a heterogeneous dose distribution into a single value which corresponds to the homogeneous dose that would give the same treatment effect.^28^ The EUD that we have used is defined as^29^

$$f^{-1}\left(\frac{1}{\left|T\right|}\sum_{i\in T}f\left(\sum_{j\in J}d_{\mathrm{ij}}t_{j}\right)\right).$$

To compare dose plans we computed the EUD, using $f\left(x\right)\;=\;x^{a}$ with *a* = −10, as used in the optimization models in Refs. 29 and 30 in dose planning for prostate cancer.

Because the dose planning in HDR BT is often performed when the patient is anesthetized, it is important to find a dose plan within a short time. Therefore, we present results for the optimization models for time limits of 3 and 15 min, and for benchmarking purposes we also present results for a time limit of 2 h.

## 4 Results

In the major part of this section, we present and compare results for the two models, DVM and DV‐MTDM, but we also give some results for model MTDM. The difference in CVaR value between the DVM and DV‐MTDM models is shown in Fig. Fig. 3, for each of the ten patients and for computing times of 3 min, 15 min and 2 h. The values in the boxplot are the differences between the results of the DV‐MTDM and the DVM models. A positive value thus means that the solution from model DV‐MTDM has a better (higher) CVaR value than the solution from model DVM. For all solution times and patients, model DV‐MTDM finds solutions which are better in terms of CVaR value, that is, dose distributions with a higher dose to the coldest volume. The reason for the large difference after 3 min is that model DVM has not yet found any nontrivial feasible solution for several patients (that is, a solution in which not all dwell times are zero). Even after 2 h, the average difference is 0.26 Gy, which is an increase of 5% with model DV‐MTDM compared to model DVM.

**Figure Fig. 3:**
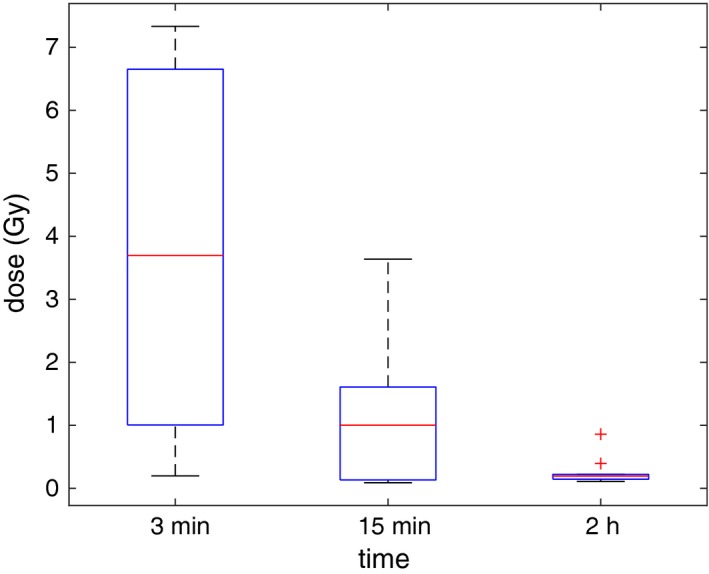
The conditional value‐at‐risk (CVaR) value for the dose‐volume mean‐tail‐dose model minus the CVaR value for the dose‐volume model for each patient, after solution times of 3 min, 15 min and 2 h, respectively. [Color figure can be viewed at http://www.wileyonlinelibrary.com]

To study how much it is possible to improve the value of CVaR we used model MTDM (see Table 2). The Gurobi solver is always able to find and prove optimality for this model (within a short time), and thus this result is an upper bound on the attainable value of CVaR. The differences between the results from model DV‐MTDM and this upper bound on CVaR are very small, and on average within only 0.1%. The upper bound on CVaR from model MTDM holds for the dose points used in the optimization model, but is not valid for the dose points used for evaluation of the obtained dose plan. This upper bound is however a strong indication that the CVaR value cannot be improved much. To significantly increase the CVaR value, relaxations of the model are necessary, by allowing a higher dose to the OAR.

Figure Fig. 4 is constructed in the same way as Fig. Fig. 3 and shows the difference in $V_{100}$ between models DV‐MTDM and DVM, for each patient and for the three computing times. Model DV‐MTDM is better in terms of $V_{100}$ for all patients after 3 min and for all but one patient after 15 min. However, after 2 h the results are very close (with an average difference in $V_{100}\;<\;1\%$).

**Figure Fig. 4:**
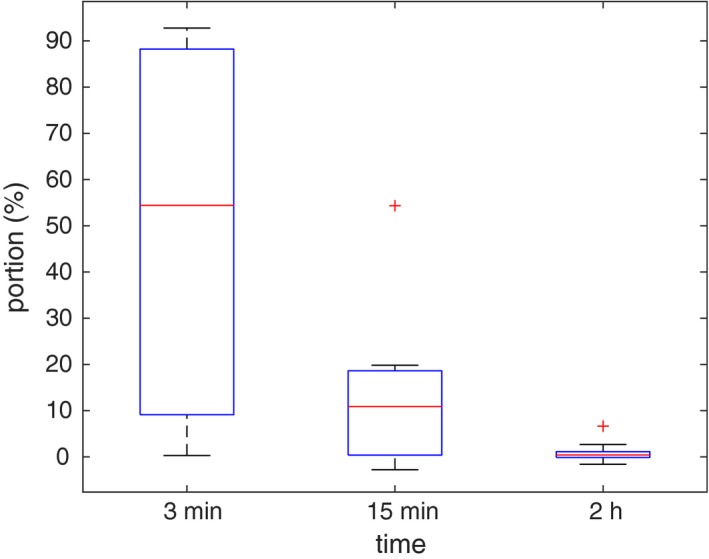
The $V_{100}$ value for the dose‐volume mean‐tail‐dose model minus the $V_{100}$ value for the dose‐volume model for each patient, after solution times of 3 min, 15 min and 2 h, respectively. [Color figure can be viewed at http://www.wileyonlinelibrary.com]

To test for statistical significance of the differences in CVaR and $V_{100}$ we used the Wilcoxon signed‐rank test.^31^ Still after 2 h, CVaR was significantly better using model DV‐MTDM (*P* = 0.002) while there was no significant difference in $V_{100}$ (*P* = 0.38).

Figures Fig. 5 and Fig. 7 show the correlation between $V_{100}$ (on the *x‐axis*), and EUD and TCP (on the *y‐axis*) respectively. The corresponding Figs. Fig. 6 and Fig. 8 show the correlation between CVaR (on the *x‐axis*), and EUD and TCP (on the *y‐axis*), respectively. The used models are DVM (marked with “x”), DV‐MTDM (marked with “+”) and MTDM (marked with “*”) and an additional model that was used only to generate dose plans for this comparison (marked with “o”). The latter model has constraints on all DIs (including $V_{100}$) and its objective is to minimize the dose to a selected subset of dose points of the PTV. The only purpose of this model was to generate dose plans with significant underdosage. In Figs. Fig. 5, Fig. 6, Fig. 7, Fig. 8, there is a data point for each of the ten patients and for each of the four models, after a solution of 2 h. These figures are meant to illustrate a trend for which evaluation criteria are closely correlated. Comparison of Figs. Fig. 5 and Fig. 7 with Figs. Fig. 6 and Fig. 8 clearly indicates that there is a higher correlation between CVaR and the two radiobiological indices than between $V_{100}$ and the radiobiological indices. This finding supports the inclusion of the CVaR component in the optimization model. Moreover, in Fig. Fig. 7 there are examples of dose plans with equal $V_{100}$ values but with quite different TCP values, and also dose plans with equal TCP values but with quite different $V_{100}$ values. There are also examples of single patients for which it is possible to improve the dose plan with respect to $V_{100}$ while ending up with a dose plan that is worse with respect to a radiobiological index (TCP value). An example of this is the red (larger) points in Fig. Fig. 7.

**Figure Fig. 5:**
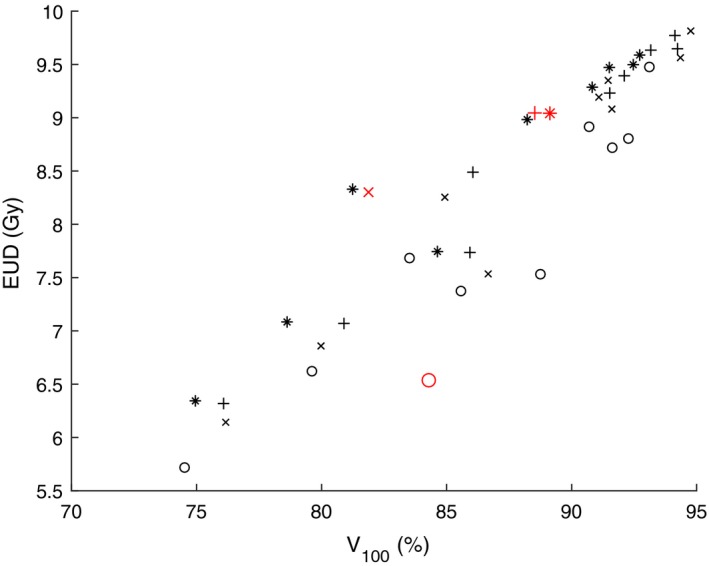
Values of $V_{100}$ and equivalent uniform dose from dose plans for the ten patients and for four models each. The red (larger) markers correspond to dose plans from one particular patient. [Color figure can be viewed at http://www.wileyonlinelibrary.com]

**Figure Fig. 6:**
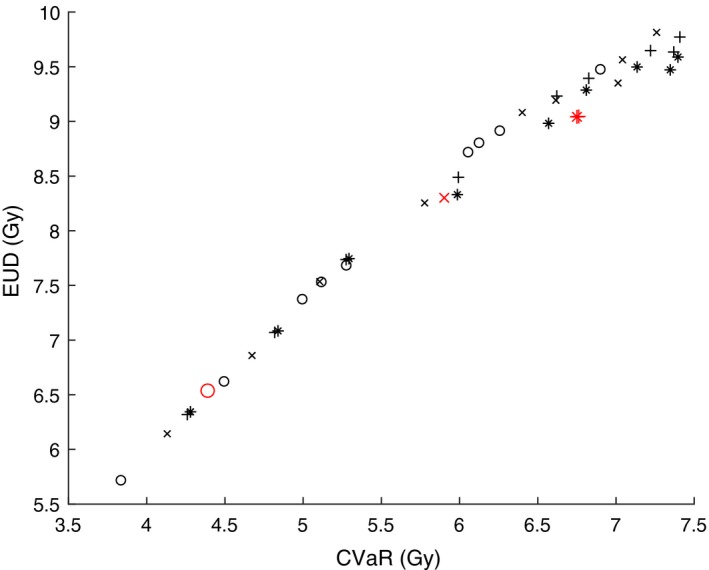
Values of conditional value‐at‐risk and equivalent uniform dose from dose plans for the ten patients and for four models each. The red (larger) markers correspond to dose plans from one particular patient. [Color figure can be viewed at http://www.wileyonlinelibrary.com]

**Figure Fig. 7:**
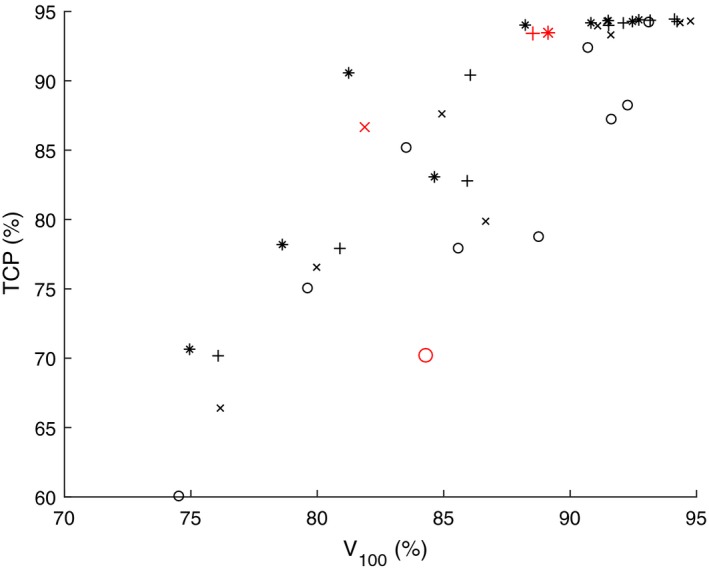
Values of $V_{100}$ and tumour control probability from dose plans for the ten patients and for four models each. The red (larger) markers correspond to dose plans from one particular patient. [Color figure can be viewed at http://www.wileyonlinelibrary.com]

**Figure Fig. 8:**
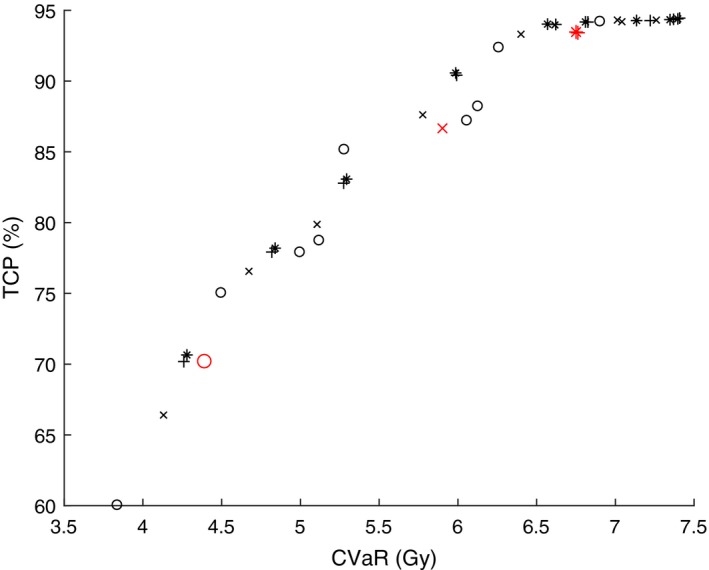
Values of conditional value‐at‐risk and tumor control probability from dose plans for the ten patients and for four models each. The red (larger) markers correspond to dose plans from one particular patient. [Color figure can be viewed at http://www.wileyonlinelibrary.com]

The solution progress for CVaR for the DVM and DV‐MTDM models is shown in Fig. Fig. 9, on a logarithmic scale. The plotted values are averages over the ten patients. At each time, model DV‐MTDM yields better results and the dose to the coldest volume of the PTV is increased, which was the aim with the model. The largest improvements can, however, be seen after a short solution time because model DV‐MTDM yields good solutions much faster than model DVM. Further, the largest improvements are found in solution times which are clinically relevant.

**Figure Fig. 9:**
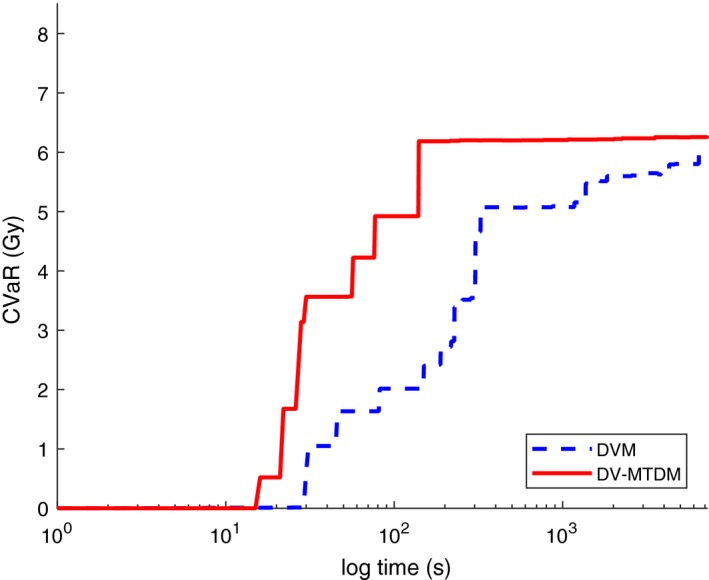
Comparison of conditional value‐at‐risk values from the dose‐volume model (dashed blue line) and the dose‐volume mean‐tail‐dose model (solid red line) with respect to solution times (logarithmic scale). [Color figure can be viewed at http://www.wileyonlinelibrary.com]

Because $V_{100}$ is a primary criterion in the clinical guidelines, we have also compared the results with respect to $V_{100}$, see Fig. Fig. 10. It can be noted that model DV‐MTDM yields the best result at all times, even though the results from models DVM and DV‐MTDM are very close after 2 h. Further, the solutions from the model DV‐MTDM are near‐optimal within solution times of minutes. It is worth noticing that even though model DV‐MTDM puts more emphasis on the coldest volume of the PTV, $V_{100}$ does not become worse.

**Figure Fig. 10:**
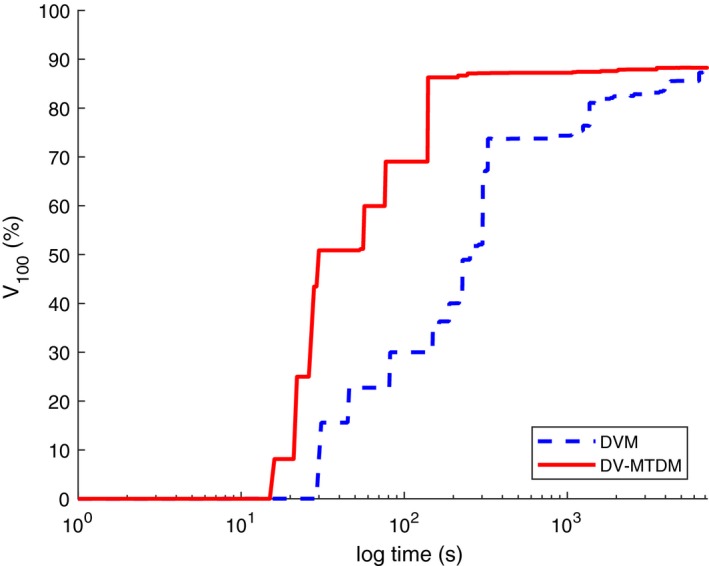
Comparison of $V_{100}$ values from the dose–volume model (dashed blue line) and the dose–volume mean‐tail‐dose model (solid red line) with respect to solution times (logarithmic scale). [Color figure can be viewed at http://www.wileyonlinelibrary.com]

Table 4 shows mean values of eight evaluation criteria at solution times 3 min, 15 min and 2 h. After 2 h, the main differences are in CVaR, EUD and $V_{100}^{\mathrm{urethra}}$. Both CVaR and EUD are significantly improved with model DV‐MTDM. The dose to urethra is consistently a little higher with model DV‐MTDM, but the result is still well within the region defined as feasible in the optimization model. The dosimetric index for the rectum is not mentioned in the table because the lower dose bound were satisfied in almost all dose points for all patients and for both models. Other attributes, including the part of the PTV where the dose is too high ($V_{150}$ and $V_{200}$), are not significantly different between the models. Also the dose homogeneity index,^32^ which is defined as

$$\frac{V_{100}-V_{150}}{V_{100}},$$

is very close between the models after 2 h.

**Table 4:** Mean values for the ten patients and the two models, for the evaluation criteria listed in the top row. Here, the letter “a” means that the results on that row are from the dose‐volume model, while “b” means that the results are from the dose‐volume mean‐tail‐dose model

	$V_{100}$ (%)	*CVaR* (Gy)	*TCP* (%)	*EUD* (Gy)	$V_{100}^{\mathrm{urethra}}$ (%)	$V_{150}$ (%)	$V_{200}$ (%)	$D_{90}$ (Gy)
3 min${}^{a}$	36	2.4	38	3.5	100	12	4.3	3.5
3 min${}^{b}$	86	6.2	88	8.5	98	27	11	8.1
15 min${}^{a}$	74	5.1	77	7.2	98	22	8.3	7.2
15 min${}^{b}$	87	6.2	89	8.5	97	27	10	8.2
2 h${}^{a}$	87	6	87	8.4	97	27	9.8	8.2
2 h${}^{b}$	88	6.3	89	8.6	93	28	10	8.3

Results in terms of $V_{100}$ and CVaR for models DVM and DV‐MTDM, after 3 min, 15 min and 2 h, are shown in Table 5. There is a wide spread in the results after 3 min, because either model DVM has not found a nontrivial feasible solution, or the best found solution is still poor. The difference is smaller after 15 min, but there are some patients for whom model DVM has still not yet found a near‐optimal solution. Finally, the results after 2 h are much closer, but the CVaR value is still improved for all patients with model DV‐MTDM compared to model DVM.

**Table 5:** The table shows, for each patient, results in terms of $V_{100}$ and CVaR for the dose–volume model (DVM) and the dose–volume mean‐tail‐dose model (DV‐MTDM), after solution times of 3 min, 15 min and 2 h respectively

		3 min		15 min		2 h	
		$V_{100}$ (%)	*CVaR* (Gy)	$V_{100}$ (%)	*CVaR* (Gy)	$V_{100}$ (%)	*CVaR* (Gy)
1	DVM	0	0.0	82	5.9	82	5.9
	DV‐MTDM	88	6.7	88	6.7	89	6.8
2	DVM	72	5.9	77	6.0	95	7.3
	DV‐MTDM	92	7.3	92	7.3	93	7.4
3	DVM	57	3.9	85	5.1	87	5.1
	DV‐MTDM	81	5.2	86	5.3	86	5.3
4	DVM	0	0.0	37	3.0	92	6.4
	DV‐MTDM	88	6.5	91	6.6	92	6.6
5	DVM	72	3.8	76	4.1	76	4.1
	DV‐MTDM	74	4.2	76	4.3	76	4.3
6	DVM	81	4.6	80	4.7	80	4.7
	DV‐MTDM	81	4.8	81	4.8	81	4.8
7	DVM	0	0.0	77	5.7	91	7.0
	DV‐MTDM	93	7.3	93	7.3	94	7.4
8	DVM	0	0.0	71	5.2	91	6.6
	DV‐MTDM	90	6.8	90	6.8	92	6.8
9	DVM	82	6.0	94	6.9	94	7.0
	DV‐MTDM	91	7.0	91	7.0	94	7.2
10	DVM	0	0.0	66	4.1	85	5.8
	DV‐MTDM	84	6.0	84	6.0	86	6.0

While the primary aim is to increase the dose to the coldest part of the PTV, it is also important to maintain a low dose to OAR. Results showing that the latter aim is achieved are presented in Table 6. Neither the DIs for urethra and rectum nor the DIs for overdosage of the PTV are significantly different. As the DVM has previously been shown to produce clinically acceptable dose plans,^8^, ^10^ these results also indicate that our extension of the DVM yields clinically relevant dose plans.

**Table 6:** Shows results after 2 h of computing time for the dose‐volume model (DVM) and the dose–volume mean‐tail‐dose model (DV‐MTDM). The dosimetric index $D_{90}$ is the lowest dose to 90% of the planning target volume. The other dosimetric indices of the same type is for organs at risk, where notation *u* and *r* is for the urethra and rectum, respectively. For these, the volume is given in cubic centimetres (cc)

		*EUD* (Gy)	$V_{150}$ (%)	$V_{200}$ (%)	$D_{90}$ (Gy)	$D_{0.1}^{u}$cc (Gy)	$D_{2}^{r}$cc (Gy)	$D_{0.1}^{r}$cc (Gy)
1	DVM	8.3	19	8	8.1	9.7	3.5	4.5
	DV‐MTDM	9.0	22	9	8.4	9.9	3.8	4.7
2	DVM	9.8	30	10	9.0	9.8	3.1	4.3
	DV‐MTDM	9.6	26	9	8.8	9.8	3.0	4.1
3	DVM	7.5	35	12	8.2	9.8	3.2	4.6
	DV‐MTDM	7.7	32	14	8.1	9.8	3.2	4.5
4	DVM	9.1	29	10	8.7	9.9	3.4	4.8
	DV‐MTDM	9.2	29	10	8.7	9.9	3.4	4.8
5	DVM	6.1	32	14	6.9	9.7	3.0	4.3
	DV‐MTDM	6.3	31	13	6.9	9.8	3.0	4.2
6	DVM	6.9	27	10	7.2	9.5	3.1	4.4
	DV‐MTDM	7.1	29	11	7.4	9.9	3.0	4.1
7	DVM	9.4	23	8	8.6	9.5	3.3	4.6
	DV‐MTDM	9.8	28	9	8.9	9.9	3.3	4.7
8	DVM	9.2	30	10	8.6	9.7	3.7	5.1
	DV‐MTDM	9.4	31	11	8.7	9.9	3.7	5.1
9	DVM	9.6	24	7	9.0	9.8	3.9	5.3
	DV‐MTDM	9.6	24	8	9.0	9.9	3.9	5.2
10	DVM	8.3	24	9	8.0	9.7	3.8	5.0
	DV‐MTDM	8.5	26	10	8.1	9.8	3.7	4.8

DVH curves are used clinically to visually inspect a dose distribution. Fig. Fig. 11 shows the DVH curves for one patient after solution times of 15 min and 2 h, respectively. The volume that receives a specific dose is at least as large for model DV‐MTDM for all dose levels. The difference is largest for dose levels between approximately 6 and 15 Gy, which includes the dose range which is specifically addressed with the CVaR component. In both figures, the curves become very similar for high dose levels.

**Figure Fig. 11:**
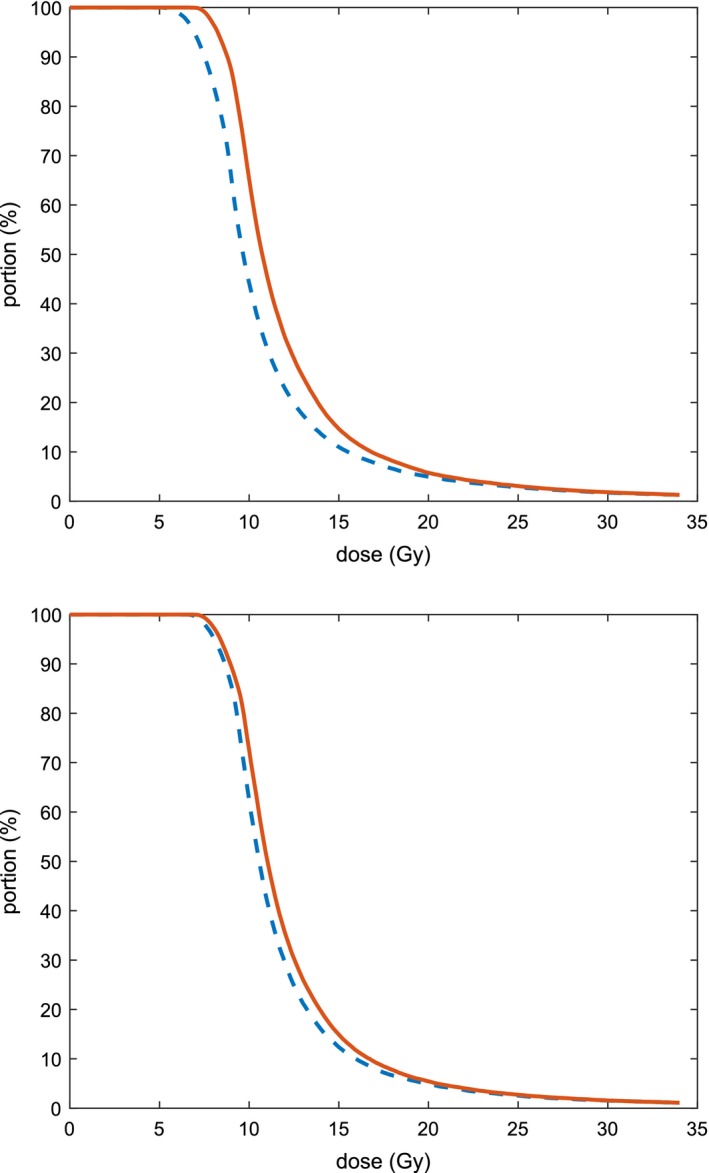
Dose–volume histograms for one patient. The dashed blue line and the solid red line show results for the dose–volume model and the dose–volume mean‐tail‐dose model respectively. The results in the top figure are obtained after 15 min, while the results in the bottom figure are obtained after 2 h. [Color figure can be viewed at http://www.wileyonlinelibrary.com]

Our solution method is the branch‐and‐bound implementation in the Gurobi solver, which is not deterministic. It is known that the performance variability is large in nondeterministic MIP solvers.^33^, ^34^ To study the stability and the predictability of the results from the solver, we performed simulations where we only varied the random seed given to Gurobi. The results from model DV‐MTDM were very stable and good solutions were found within a short time for all patients, regardless of which random seed that was used. Model DVM showed a much larger variation in both the time to the first (nontrivial feasible) dose plan and in the solution progress.

## 5 Discussion

We have presented results for models DVM and DV‐MTDM with one set of parameter values such as prescription dose and dose bounds. For some patients, the values of $V_{100}$ and $D_{90}$ are lower than those suggested in the guidelines,^3^ but the results with our choices of parameter values should still be valid for the sole purpose of comparing the models.

We have also performed computer simulations with other parameter values. These simulations show similar overall behavior and are therefore not included.

To get an indication of the adverse treatment effects from cold volumes, we have used the radiobiological evaluation criteria TCP and EUD. Because of the difficulty in estimating the values of their parameters (particularly for TCP), the absolute values are rather uncertain and not a factual representation of the treatment effect. To move from using DIs for evaluating dose plans to using radiobiological measures as the primary evaluation criteria, more research is needed.^26^

In Ref. 29, tail EUD was introduced in an optimization model for IMRT. This measure is analogous to EUD but only takes the dose to the coldest volume in the PTV into account. The resulting optimization model is nonlinear, but convex. The aim with this model was to reduce the underdosed volume, which is similar to the aim of the model proposed in this paper. However, by instead using the linear CVaR measure for reducing cold volumes, the model remains linear and our extended MIP model can be solved with the methods that have been used to solve the original MIP model DVM. This would not be the case if we had used tail EUD for reducing cold volumes, because it is nonlinear.

Optimizing dose plans only with respect to DIs, the doses to the coldest volume of the structure (in case of the PTV) are not taken into account at all. Further, whenever mathematical optimization is used in dose planning, the optimization process is guided solely by the components and aspects included in the model. Because any optimization process exploits the degrees of freedom that are available in the model, any oversight to include relevant modeling components can have a very adverse effect on the outcome of the model. This is true also for the DVM, and hence there is a risk that the treatment effect is lower than intended, as measured by only the DI. Further studies are needed to see to what extent this would be a problem in clinical practice.

## 6 Conclusions and future research

The motivation for our study was to address the weakness of the DVM that underdosage to small target volumes is not at all taken into account in treatment planning. The study by Tomé and Fowler^14^ on the adverse effect of underdosage motivates the increased priority to the dose to cold volumes.

The DVM has received great interest in the last decade and has been designed to explicitly include the criteria of clinical importance. Our model comparison was carried out with previous studies on clinical feasibility of the DVM in mind.^8^, ^10^ However, in these studies, the dose to the coldest part of the PTV was not analysed.

Our proposed improved DVM achieved its aim to increase the dose to the coldest volume of the PTV, while at the same time keeping $V_{100}$ at a level that is comparable to that obtained from the DVM. Furthermore, the dose to the coldest volume was shown to be very close to the best attainable value. This observation implies that we have to relax the DI constraints on the OAR to be able to further increase the dose to the coldest volume. The improved model also consistently provided near‐optimal solutions much faster than the standard DVM model. This improvement was especially seen for clinically relevant solution times (of <15 min).

For clinical usage it is important to be able to foresee how much computing time that is needed to find a (reasonably) good solution. The stability of the model DV‐MTDM in this respect is an important advantage of this model. In particular, using model DV‐MTDM, near‐optimal solutions were always found within 3 min for all patients, while when using model DVM this was the case only for half of the patients. It was further observed that the MIP solver was not even able to find nontrivial feasible solutions of the DVM within 3 min for all patients; this is consistent with the results in Ref. 26. (We observed similar patterns when varying the random seed in the MIP solver Gurobi.)

Dose planning in HDR BT has traditionally been a manual task. In manual planning there are often implicit criteria and considerations, for example, to avoid cold volumes or hot spots. The usage of mathematical optimization, which is an automatic way to construct dose plans based on more or less simplified models, may yield dose distributions that are fundamentally different from the dose distributions from manual planning. This is both because only criteria that are explicitly included in the optimization model are considered and because the nature of optimization methodologies is such that they tend to give more extreme solutions. There is therefore a need to investigate further which criteria that should be used in mathematical optimization models for dose planning, possibly resulting in different guidelines than for manual planning.

Because the solution times for the studied optimization models depend on the solution method, it would be interesting to study if other methods for solving the proposed models, such as heuristics, would still give shorter solution times for model DV‐MTDM compared to model DVM.

Finally, the aim of this paper was to evaluate our extended formulation of the DVM against the standard formulation of the DVM. An important topic for future research is to study the features of our extended optimization model for dose planning from a clinical perspective. Future work is to compare the proposed model with both models for automatic dose planning used in existing clinical treatment planning systems, and manual planning, and combinations of these.
